# Supplementary material for: Daily Life Changes and Life Satisfaction among Korean School-Aged Children in the COVID-19 Pandemic
Source: Int J Environ Res Public Health. 2021 Mar 23;18(6):3324. doi: 10.3390/ijerph18063324 (PMC8004811; doi:10.3390/ijerph18063324)
Supplement: Supplementary file 1 [file ijerph-18-03324-s001.pdf]

Supplementary material – Details of G-Power protocols

<1> Power calculation for independent *t*-test between during-COVID-19 (*n* = 166) vs. KCYWI 2019 (*n* = 334)

t tests - Means: Difference between two independent means (two groups)

**Analysis:**	Post hoc: Compute achieved power		
**Input:**	Tail(s)	=	Two
	Effect size d	=	0.5
	α err prob	=	0.05
	Sample size group 1	=	166
	Sample size group 2	=	334
**Output:**	Noncentrality parameter δ	=	5.2651686
	Critical t	=	1.9647390
	Df	=	498
	Power (1-β err prob)	=	0.9995079

<2> Power calculation for independent *t*-test between during-COVID-19 (n = 166) vs. KCYPS 2018 (n = 1,236)

t tests - Means: Difference between two independent means (two groups)

**Analysis:**	Post hoc: Compute achieved power		
**Input:**	Tail(s)	=	Two
	Effect size d	=	0.5
	α err prob	=	0.05
	Sample size group 1	=	166
	Sample size group 2	=	1236
**Output:**	Noncentrality parameter δ	=	6.0486614
	Critical t	=	1.9616599
	Df	=	1400
	Power (1-β err prob)	=	0.9999779

<3> Power calculation for multiple regression analysis on during-COVID-19 (n = 158)

F tests - Linear multiple regression: Fixed model, R^2 deviation from zero

**Analysis:**	Post hoc: Compute achieved power		
**Input:**	Effect size f^2	=	0.15
	α err prob	=	0.05
	Total sample size	=	158
	Number of predictors	=	4
**Output:**	Noncentrality parameter λ	=	23.7000000
	Critical F	=	2.4307718
	Numerator df	=	4
	Denominator df	=	153
	Power (1-β err prob)	=	0.9823659
